# Case report: Infection-associated HPS during pregnancy cured by HLH-94 protocol with induction therapy of ruxolitinib

**DOI:** 10.3389/fimmu.2024.1483257

**Published:** 2024-11-15

**Authors:** Tianqi Cen, Weixia Xuan, Shaohui Huang, Ziqi Wang, Lijun Shen, Moyuan Zhang, Jinzhou Fang, Shenying Yang, Xiaoju Zhang

**Affiliations:** ^1^ Department of Respiratory and Critical Care Medicine, Xinxiang Medical University, Henan Provincial People’s Hospital, Zhengzhou, China; ^2^ Department of Respiratory and Critical Care Medicine, Zhengzhou University People’s Hospital, Henan Provincial People’s Hospital, Zhengzhou, China

**Keywords:** hemophagocytic syndrome, pregnancy, ruxolitinib, HLH-94, induction therapy

## Abstract

Hemophagocytic syndrome (HPS) is a rapidly progressive and highly fatal disease, and is even more complex when it occurs during pregnancy. Currently, the HLH-94 protocol is commonly used for treatment for HPS, with ruxolitinib being mostly used for salvage therapy. Here, we report a pregnant woman who presented with fever, thrombocytopenia, splenomegaly, and subsequently developed into severe pneumonia and multiple organ dysfunction(MODS). The patient was diagnosed as HPS based on clinical manifestations, laboratory indexes, and hemophagocytosis observed in bone marrow aspirate smear. After receiving ruxolitinib as induction therapy combined with HLH-94 protocol, the patient significantly improved and eventually cured.

## 1 Introduction

Hemophagocytic syndrome (HPS), also known as hemophagocytic lymphohistiocytosis (HLH), is characterized by an excessive inflammatory response caused by abnormal activation and proliferation of lymphocytes, monocytes, macrophages, as well as the inflammatory cytokines secreted, which can arise from either hereditary or acquired factors (1). HPS is rare yet highly lethal, and lacks specific clinical manifestations, for which timely diagnosis and treatment are essential (2). The global incidence of HPS is estimated to be 1/800,000 per year, with the majority of adult cases located in Asia, and HPS in pregnancy is even rarer and more dangerous (3). Currently, HPS is mostly treated with the HLH-94 protocol, which utilizes etoposide and dexamethasone for first-line treatment. In addition, ruxolitinib is also used for the therapy of HPS only when HLH was failed, which was known as salvage therapy. Here, we report a case of ruxolitinib as induction therapy combined with the HLH-94 protocol to cure infection-associated HPS in early pregnancy.

## 2 Case report

A 25-years-old female at 12 weeks pregnant was admitted to the respiratory intensive care department(RICU) of our center with fever for 7 days and thrombocytopenia for 2 days, with no specific past, personal, marital or family history. Upon admission, the patient’s physical examination revealed body temperature (T) 39.5°C, breathing 23 times/min, heart rate 148 beats/min and blood pressure 124/74 mmHg. Chest CT scan revealed patchy shadows with increased density in both lungs, which indicated inflammation in both lungs. Laboratory examination revealed thrombocytopenia, elevated C-reactive protein, elevated D-dimer, elevated transaminases, elevated bilirubin, and elevated ferritin level. Considered that the patient’s condition was critical with severe pneumonia and cardiac, respiratory, hepatic failures and coagulation dysfunction, we immediately started with meropenem as an experiential therapy and other supportive therapy, while actively seeking the underlying cause, during which the fever occurred repeatedly and up to 38.2°C. Rheumatism was firstly ruled out since relevant laboratory indicators were negative, and no symptoms such as joint swelling, pain, skin rash, or others related to rheumatism were observed. Yet hematological system diseases and infectious disease cannot be excluded. Then we performed bone marrow aspiration. Additionally, due to the severe pulmonary infection, we conducted bedside bronchoscopic examination to identify the potential pathogen. Bronchoalveolar lavage fluid (BALF) was collected and Metagenomic Next Generation Sequencing (mNGS) as well as Targeted Next Generation Sequencing(tNGS) was conducted, which revealed over-represented reads mapping to multiple pathogens, mainly aspergillus flavus and adenovirus. Based on these results, we implemented treatment included meropenem, sulfamethoxazole, voriconazole, amphotericin B, linezolid, and tigecycline for anti-infection, along with tracheal intubation, artificial liver support, plasma exchange, and symptomatic treatment.

**Figure 1:**
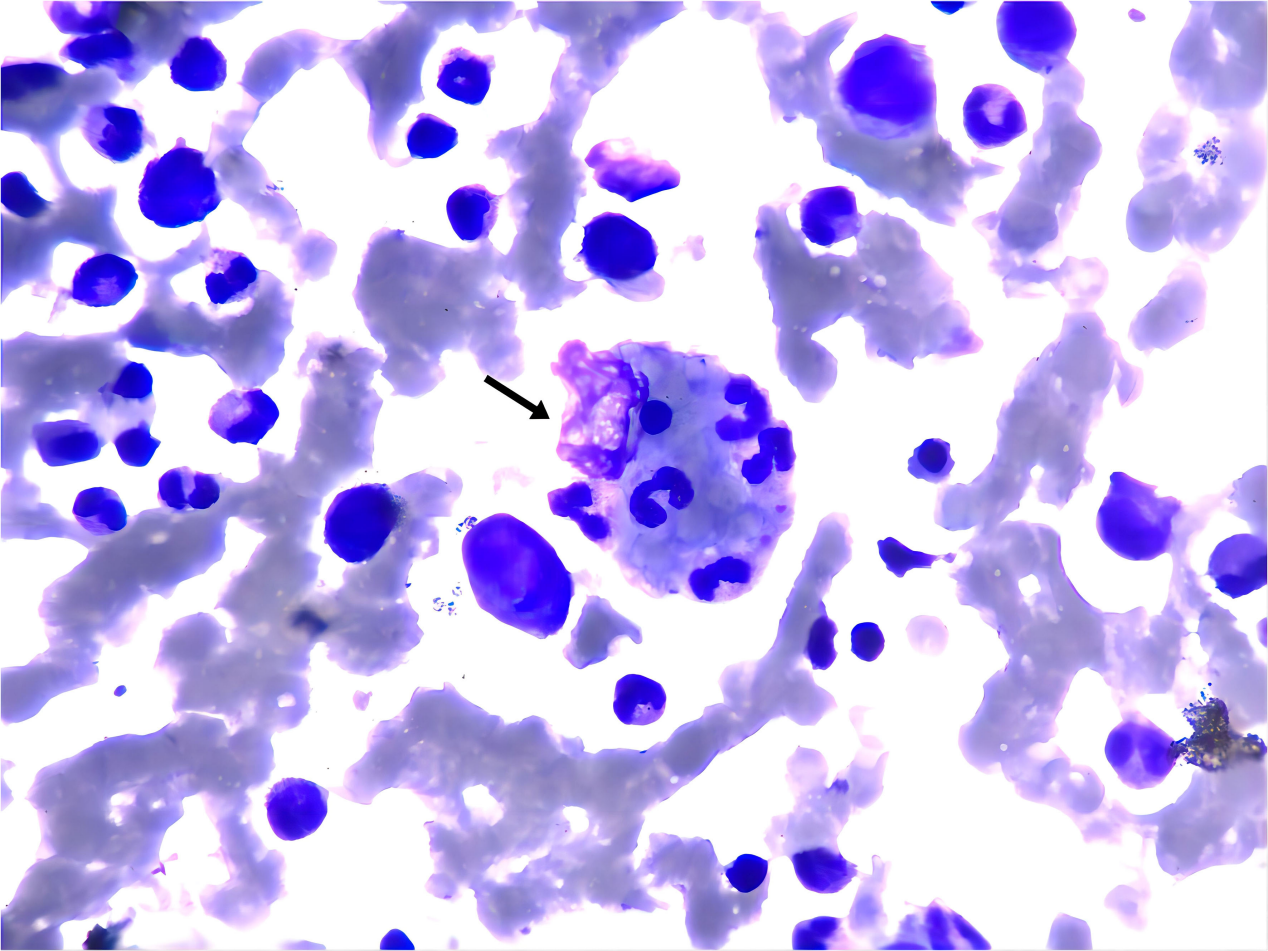
Wright-Giemsa stain of bone marrow aspirate smear (anterior superior iliac spine) showed the evidence of hemophagocytosis, Macrophage engulfing erythrocytes, platelets, and neutrophils (black arrow); original magnfication ×1000.

**Figure 2:**
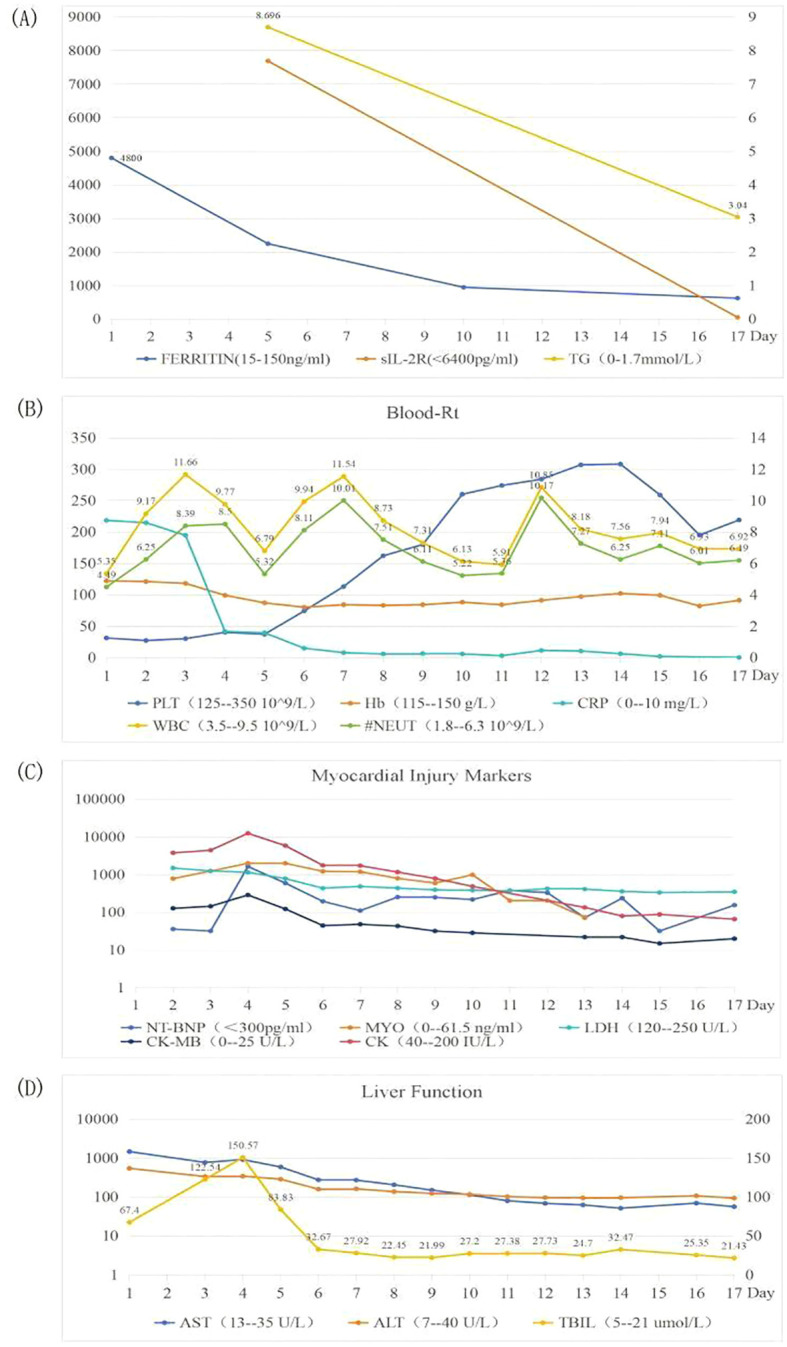
Trends of laboratory indicators. Picture **(A)** showing FERRITIN, sIL-2R and TG; picture **(B)** showing Blood-Rt; picture **(C)** showing Myocardial injury markers; picture **(D)** showing Liver Function, during hospitalization.

**Figure 3:**
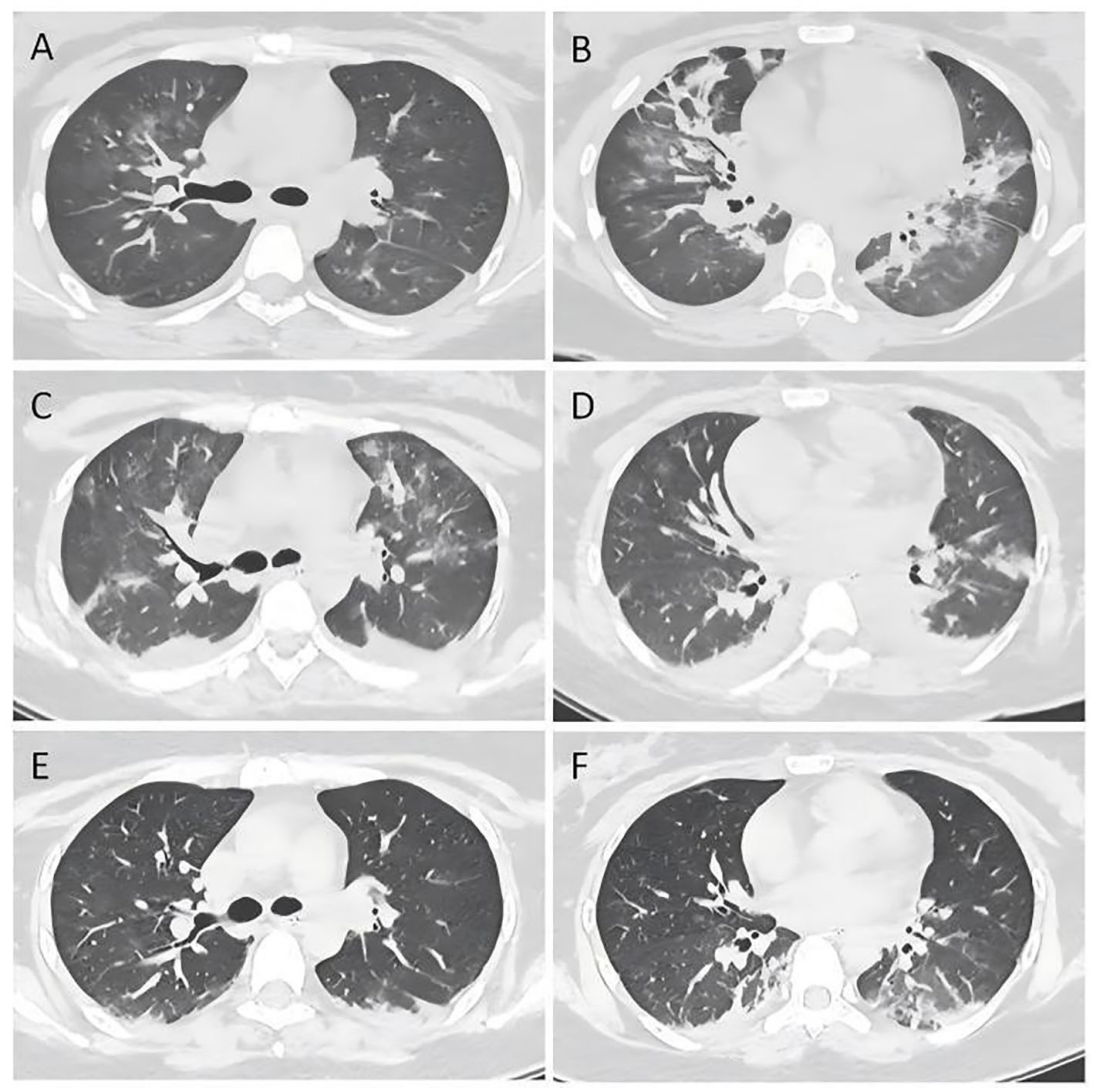
Chest computed tomography scan on day 2 **(A, B)**, day 6 **(C, D)** and day 12 **(E, F)**.

On the 4th day of admission, we obtained result of bone marrow aspiration, which revealed (**Figure 1**) active bone marrow proliferation with visible hemophagocytic cells; mature red blood cells varied in size, with an increased white blood cell count, elevated granulocyte ratio, and scarce platelets. The diagnosis of hemophagocytic syndrome (HPS) was then made based on following evidences: 1) persistent fever and splenomegaly; 2) laboratory findings including thrombocytopenia, decreased fibrinogen level, and elevated ferritin level; 3) hemophagocytic cells observed in the bone marrow aspirate smear. HLH-94 protocol would be primary choice after the diagnosis of HPS, however, the patient’s condition was critical, which preclude her from receiving chemotherapy due to potential intolerance. Since ruxolitinib was also reported to be effective for HPS with a better safety profile, we prescribed ruxolitinib at a decreased dose of 5mg Bid (standard treatment dose is 15-20mg Bid) as well as dexamethasone 20mg QD. 2 days post-treatment, the patient’s body temperature returned to normal and remained stable, and her cardiac, respiratory, hepatic, and coagulation functions gradually improved. On the 6th day of admission(that is the third day of low dose ruxolitinib treatment), although complete response [CR was defined as no fever, a normal spleen size, no cytopenia, no hyperferritinemia (defined as a ferritin level>2000 ng per milliliter), no evidence of coagulopathy, no neurologic or cerebrospinal fluid abnormalities attributable to hemophagocytic lymphohistiocytosis, and no sustained increase in the level of soluble CD25 (4)] was not achieved, we considered that the patient was ready for HLH-94 protocol after a thorough assessment, so we prescribed her regimen consisted of etoposide 100mg and changed to 150mg the next day as HLH-94 protocol indicated, and continued ruxolitinib 5mg Bid/dexamethasone 20mg QD. The patient’s condition steadily improved. On the 12th day of hospitalization, the patient’s body temperature returned to normal, all laboratory indicators demonstrated a steady trend of recovery (**Figure 2**, Daily body temperature and detailed laboratory indicators were shown in **Supplementary File**), and the pulmonary infection was alleviated (**Figure 3**). Then we stopped the prescription of ruxolitinib.

Taking into account the patient’s own wishes and the potential teratogenicity of etoposide, she was transferred to the obstetrics department for induced labor. During this period, the patient’s condition is stable and was discharged after 17 days’ admission. Afterwards, she continued to receive ruxolitinib and HLH-94 protocol and reached the CR standard in the 6th week.

After diagnosis, we also tested HPS hotspot genes (Detailed names of HPS hotspot gene were shown in **Table 1**) mutation through NGS, and no typical disease-related mutations were identified. In addition, lymph node biopsy ruled out the possibility of lymphoma, and PCR of blood further exclude the infection of EB virus or cytomegalovirus. In the end, we concluded that the HPS was caused by pregnancy combined with severe pneumonia from mixed infections of various pathogens, mainly Aspergillus flavus and adenovirus.

**Table 1:** Standardized test for HPS hotspot genes.

Names of HPS hotspot gene							
ITK	CD27	MAGT1	CD70	CTPS1	RASGRP1	CDC42	SH2D1A
XIAP	ORAI1	CD3E	IL2RG	IL7R	BTK	IFNGR1	IFNGR2
LIPA	MVK	SLC7A7	PRF1	UNC13D	STX11	STXBP2	NLRC4
NLRC3	NLRP12	NLRP13	NLRP4	NRAS	ARF6	ARHGEF6	ERCC4
G3BP1	GNLY	GZMB	IL16	LAMP1	RASGRP3	SRGN	STAT4
TREM2	LYST	RAB27A	AP3B1	PIK3CD	STAT3	NCF1	STAT1
STAT2	CARMIL2	RAG1	RAG2	WAS	CASP10	DOCK8	LRBA
MCM3AP	MCM9	TTC7A	IKBKG	CYBB	ATM	FAS	CYBA
DKC1	MEFV	TNFRSF1A	PIK3CG	HAVCR2			

## 3 Discussion

HPS is a kind of severe inflammatory syndrome resulting from inherited or acquired immune dysfunctions. It is even more complex when it occurs during pregnancy. The pathogenesis of pregnancy-related HPS is unclear, and was thought to be associated with type 2 T helper cells (Th2) dominance of Th lymphocytes during pregnancy. Type 1 T helper cells (Th1) mainly secrete pro-inflammatory cytokines that drive cell-mediated immunity (CMI), while Th2 cells was more related with antibody production, and the balance between Th1 and Th2 is crucial for the stability of the immune system. During pregnancy, there is often a skewing from Th1 to Th2, which reduces CMI of Th1 and raises susceptibility to viruses (5). Additionally, this reduction in CMI can lead to an excessive activation of hemophagocytes as the initial immune response to infection (6). Besides, pregnancy itself can also act as a contributor to HPS (3). Pregnancy itself often companied with a state of systemic inflammation, with the placenta producing a significant amount of cytokines; when this state is affected by factors such as infection, the excessive inflammatory response and cytokine storm may trigger HLH (7, 8). Furthermore, the abnormal inflammatory state caused by pregnancy can also be evidenced by some report of severe systemic inflammatory response (SIRS) and MODS related to the mother-fetus exchange during pregnancy, including HLA antigens, cytotrophoblasts, cellular debris, etc., in the fetal blood (8, 9).

The diagnostic criteria currently accepted were proposed by Histiocyte Society in 2004 (10), this patient fully meets the HLH-2004 diagnostic criteria of HPS.

The treatment for HPS primarily encompasses two components: first, therapy to control the excessive inflammatory state and mitigate HPS progression; second, etiological therapy to treat the primary disease and potential immunodeficiency, thus preventing the recurrence of HPS (11). Currently, the treatment of HPS mostly relies on HLH-94 protocol, which utilizing etoposide as first-line therapy (12–15).

Ruxolitinib, a JAKl/2 inhibitor, targets the inflammatory response in a different manner than steroids and etoposide, blocking the overproduction of inflammatory factors, is mostly used for salvage treatment of HPS (16, 17). In recent years, due to its better efficacy and safety profile, ruxolitinib has gradually been applied in the first-line treatment of HLH to rapidly improve laboratory indicators (18–22). Slostad et al. (18) used ruxolitinib for the first time in the first-line treatment of HPS. To our knowledge, this is the first case where ruxolitinib was used as the induction therapy before the HLH-94 regimen, for infection-associated HPS during pregnancy, resulting in a cure. In our case, although treatment with ruxolitinib did not reached CR, it created suitable physical conditions for subsequent chemotherapy. Therefore, we suggest that when the patient is temporally intolerant with chemotherapy, ruxolitinib may be a good choice as an induction therapy before chemotherapy.

Despite refinements in treatment plans, the mortality rate for HPS patients during pregnancy remains high, and maternal and fetal mortality rates can be as high as 22% and 40%, respectively (8). Infection is a major cause of HPS in pregnant women, for pregnancy with fever, hematopenia, severe pneumonia and MODS, who show no significant improvement after treatment, the possibility of HPS should be considered in time to shorten diagnostic time and improve the prognosis. For patients with HPS in pregnancy, when chemotherapy cannot be tolerated, ruxolitinib can be a choice as induction treatment to create an opportunity to receive HLH-94 protocol, and further study is warranted.

## 4 Conclusion

Hemophagocytic syndrome (HPS) is a life-threatening disease, especially when it occurs during pregnancy. We present the case of an pregnant woman with infection-associated HPS, who presented with fever, thrombocytopenia, splenomegaly, and subsequently developed into severe pneumonia and multiple organ dysfunction(MODS). The greatest barrier to the early treatment of HLH in this patient was temporally intolerant with chemotherapy. After receiving ruxolitinib as induction therapy combined with HLH-94 protocol, the patient significantly improved and eventually cured.

## Data Availability

The original contributions presented in the study are included in the article/**Supplementary Material**. Further inquiries can be directed to the corresponding author.
