# Supplementary material for: Case report: Infection-associated HPS during pregnancy cured by HLH-94 protocol with induction therapy of ruxolitinib
Source: Front Immunol. 2024 Nov 15;15:1483257. doi: 10.3389/fimmu.2024.1483257 (PMC11604626; doi:10.3389/fimmu.2024.1483257)
Supplement: Supplementary file 1 [file Table1.docx]

| Day | T | FERRITIN  (15-150ng/ml) | sIL-2R  (<6400pg/ml) | TG  (0-1.7 mmol/L) | D-D  (0 - 0.55 μg/ml) | FBG  (1.8--3.5 g/L) | Blood-Rt | | | | |  | Myocardial Injury Markers | | | | |  | Liver Function | | |
| --- | --- | --- | --- | --- | --- | --- | --- | --- | --- | --- | --- | --- | --- | --- | --- | --- | --- | --- | --- | --- | --- |
|  |  |  |  |  |  |  | PLT  (125 - 350 10^9/L) | Hb  (115--150 g/L) | WBC  (3.5--9.5 10^9/L) | #NEUT  (1.8 - 6.3 10^9/L) | CRP  (0 - 10 mg/L) |  | NT-BNP  (<300pg/ml) | MYO  (0--61.5 ng/ml) | LDH  (120--250 U/L) | CK  (40 - 200 IU/L) | CK-MB  (0 - 25 U/L) |  | AST  (13--35 U/L) | ALT  (7 - 40 U/L) | TBIL  (5--21 umol/L) |
| 1 | 38.5 | 4800 | - | - | 80.91 | 2.1 | 31 | 122 | 5.35 | 4.49 | 218.32 |  | - | - | - | - | - |  | 1454.3 | 540.5 | 67.4 |
| 2 | 38.2 | - | - | - | 34.18 | 1.7 | 27 | 121 | 9.17 | 6.25 | 214.68 |  | 36 | 782.9 | 1496 | 3781 | 128 |  | - | - | - |
| 3 | 36.9 | - | - | - | 34.39 | 1.36 | 30 | 118 | 11.66 | 8.39 | 194.5 |  | 32 | 1238 | 1259 | 4440 | 145 |  | 766.8 | 333.1 | 122.54 |
| 4 | 38.2 | - | - | - | 27.47 | 1.27 | 40 | 99 | 9.77 | 8.5 | 41.44 |  | 1640 | 2000 | 1146 | 12447 | 289 |  | 909.4 | 340.2 | 150.57 |
| 5 | 36.4 | 2246 | 7688.15 | 8.696 | 6.79 | - | 37 | 87 | 6.79 | 5.32 | 39.17 |  | 593 | 2000 | 783.9 | 5851.8 | 122.73 |  | 586.2 | 285 | 83.83 |
| 6 | 36.5 | - | - | - | 1.9 | 1.42 | 74 | 80 | 9.94 | 8.11 | 14.71 |  | 196 | 1222 | 437.4 | 1763 | 44.65 |  | 273.4 | 157.5 | 32.67 |
| 7 | 36.7 | - | - | - | 1.82 | 1.4 | 113 | 84 | 11.54 | 10.01 | 7.62 |  | 111 | 1193 | 485.7 | 1724.4 | 48.16 |  | 269.7 | 160.9 | 27.92 |
| 8 | 37.1 | - | - | - | 2.42 | 1.69 | 162 | 83 | 8.73 | 7.51 | 5.53 |  | 254 | 796.2 | 438.9 | 1169.6 | 43.38 |  | 204.5 | 136.4 | 22.45 |
| 9 | 36.9 | - | - | - | 1.78 | 1.65 | 180 | 84 | 7.31 | 6.11 | 6.14 |  | 249 | 595.6 | 394.7 | 790.8 | 31.97 |  | 148.9 | 123.1 | 21.99 |
| 10 | 36.9 | 947.7 | - | - | 1.45 | 1.5 | 260 | 88 | 6.13 | 5.22 | 5.55 |  | 219 | 990.1 | 384.8 | 486.7 | 28.59 |  | 113.2 | 116.7 | 27.2 |
| 11 | 37.1 | - | - | - | 1.48 | 2.18 | 274 | 84 | 5.91 | 5.36 | 2.77 |  | 378 | 204.3 | 370.6 | - | - |  | 79.4 | 101.4 | 27.38 |
| 12 | 36.7 | - | - | - | 1.24 | 2.24 | 284 | 91 | 10.85 | 10.17 | 11.15 |  | 332 | 202.9 | 422 | - | - |  | 68.1 | 95.3 | 27.73 |
| 13 | 36.9 | - | - | - | 1.06 | 2.18 | 307 | 97 | 8.18 | 7.27 | 10.21 |  | 72 | 74.1 | 413.1 | 134.7 | 22.06 |  | 62.1 | 93.9 | 24.7 |
| 14 | 36.6 | - | - | - | 1.26 | 1.88 | 308 | 102 | 7.56 | 6.25 | 5.95 |  | 236 | - | 360 | 80 | 22 |  | 51.1 | 95 | 32.47 |
| 15 | 36.5 | - | - | - | - | - | 259 | 99 | 7.94 | 7.11 | 1.58 |  | 32 | - | 336 | 88 | 15 |  | - | - | - |
| 16 | 36.8 | - | - | - | - | - | 195 | 82 | 6.93 | 6.01 | - |  | - | - | - | - | - |  | 69.1 | 106.3 | 25.35 |
| 17 | 36.6 | 623 | 55.27 | 3.04 | - | - | 219 | 91 | 6.92 | 6.19 | <0.5 |  | 155 | - | 348 | 66 | 20 |  | 55.9 | 93 | 21.43 |

Supplementary File. Daily body temperature and laboratory indicators
